# Spinal Deformities and Advancement in Corrective Orthoses

**DOI:** 10.3390/bioengineering8010002

**Published:** 2020-12-25

**Authors:** Athar Ali, Vigilio Fontanari, Marco Fontana, Werner Schmölz

**Affiliations:** 1 Department of Industrial Engineering, University of Trento, 38122 Trento, Italy; vigilio.fontanari@unitn.it (V.F.); marco.fontana-2@unitn.it (M.F.); 2 Department of Trauma Surgery, Medizinische Universität Innsbruck, 6020 Innsbruck, Austria; werner.schmoelz@i-med.ac.at

**Keywords:** braces, scoliosis, corrective orthosis, wearable robotics, rehabilitation robotics

## Abstract

Spinal deformity is an abnormality in the spinal curves and can seriously affect the activities of daily life. The conventional way to treat spinal deformities, such as scoliosis, kyphosis, and spondylolisthesis, is to use spinal orthoses (braces). Braces have been used for centuries to apply corrective forces to the spine to treat spinal deformities or to stabilize the spine during postoperative rehabilitation. Braces have not modernized with advancements in technology, and very few braces are equipped with smart sensory design and active actuation. There is a need to enable the orthotists, ergonomics practitioners, and developers to incorporate new technologies into the passive field of bracing. This article presents a review of the conventional passive braces and highlights the advancements in spinal orthoses in terms of improved sensory designs, active actuation mechanisms, and new construction methods (CAD/CAM, three-dimensional (3D) printing). This review includes 26 spinal orthoses, comprised of passive rigid/soft braces, active dynamics braces, and torso training devices for the rehabilitation of the spine.

## 1. Introduction

Over 600,000 patients with spinal deformity are treated every year [1]. A spine deformity, such as kyphosis and idiopathic scoliosis, is an abnormality in the spine curvature. Diseases instigated by spinal stenosis, spondylolisthesis, and vertebral fractures, also result in spine deformity [2]. Spine deformity limits daily life activities and can damage the musculoskeletal, respiratory, and nervous systems [1]. The conventional treatment of spinal deformity is bracing, with the main objective to restrict the cobb angle progression and palliate the inevitability for surgery [3]. In the early 20th century, the use of braces was reduced due to surgical intervention, until the mid-20th century when complications started to emerge in spinal surgeries. This drew attention back to the conventional brace treatment [4].

Several literature studies [4,5,6,7,8,9,10,11,12] have been conducted to evaluate the effectiveness of corrective orthoses. These studies mainly considered the selection of the effective brace type and predictive factors (compliance, curve magnitude, growth stage, body mass index, and exercise potential) that contribute to promising outcomes.

This article presents a review of corrective orthoses to treat spinal deformities. It mainly focuses on developments in spinal orthosis technology, such as material, construction, rigidity, actuation, sensing, and characterization of different mechanical parameters. The devices developed for rehabilitation and training of the torso have also been reviewed in this study. The objective is to highlight advancements in brace technology and enable ergonomics practitioners, orthotists, and developers to design corrective orthosis for the treatment of injuries and deformities of the spine column.

## 2. Materials and Methods

**Figure 1:**
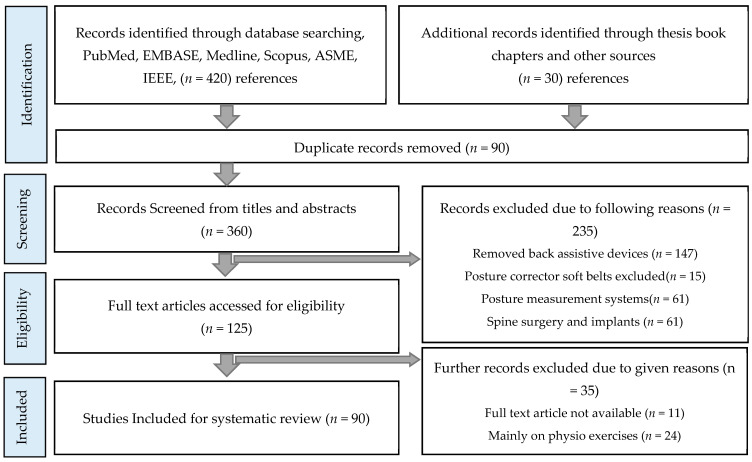
Systematic review article selection flowchart.

This systematic review on corrective orthosis was compiled using PRISMA guidelines. Records have been identified by searching through various research databases, such as PubMed, Medline, CINHAL, PEDro, EMBASE, Scopus, IEEE digital library, ASME digital library, and additional records through other sources. Initial searches showed 450 research references, which were further reduced to 360 by eliminating duplicate records. The remaining references were then screened by titles and abstracts to shortlist 125 references. References were excluded due to the reasons given in Figure 1. The remaining 90 references were considered eligible, and were then fully reviewed and included in the paper. Records that describe planning stages and have not yet taken physical form are not included in this review. This review includes references from journals, conferences, and commercially developed devices. There are a total of 26 devices mentioned in this study: 14 rigid and 5 soft braces, as well as seven other spinal rehabilitation and training devices.

Several keywords and a combination of medical subject heading terms, such as braces, spinal orthosis, conservative treatment, scoliosis, spinal deformities, and rehabilitation robotics were used. Several data variables were considered while compiling this review, such as construction, structure, and correction principle.

## 3. Corrective Orthoses (Braces)

Braces have been used for centuries to treat spinal deformities like scoliosis, kyphosis, and lordosis. Many braces were developed in the mid-20th century and can be classified based on their construction, rigidity, symmetry, and openings (posterior/interior), as well as the principle of correction [13]. Some braces are constructed to apply de-rotation and tractive force to the spine [14] or pure spine bending [15], while others are custom-made to provide three-point pressure bending along with de-rotation on abnormal spine curves and apices [16,17].

The concept of bracing to treat scoliosis reattracted people’s attention in the middle of the 20th century, due to an increase in complications in surgical treatment. Several rigid braces, such as Milwaukee [14,18,19,20,21], Boston [22,23,24,25], Lyon [26,27,28], and Chêneau braces [29,30,31,32], were developed for treating different scoliosis curves and have different correction principles.

To achieve better corrective results, hard braces need to be worn over 18 h a day, which seriously affects the activities of daily life. Therefore, nighttime braces were developed to reduce the wear time and enhance the compliance of the wearing braces. The Charleston brace [33] and the Providence brace [15] are two prominent nighttime braces. The Charleston brace is a part-time brace, effective for the patients with single thoracolumbar, single-bending scoliosis, and needs to be worn 8–10 h every night [6]. It has an aggressive over-correcting mechanism, and it keeps the patient’s posture correct through lateral forces. Although rigid braces reduce the cobb angle significantly, a deep knowledge of curve pattern identification, basic biomechanics, and an understanding of functional diagnosis is needed for technicians to apply this brace.

Rigid braces are quite effective in the treatment of spinal curves. However, due to the static and rigid nature of the braces, they weaken the muscles around the spine and lead to other spine complexities. Although rigid braces are considered to be more effective in curve correction, considerable shortcomings are present in the current rigid braces: (i) braces limit motion, resulting in weakening of the muscles around the spine; (ii) they affect cardiopulmonary efficiency; (iii) they do not comprehend precise force control over a specific vertebra; (iv) braces are not modulated according to users’ needs; (v) long construction time; (vi) braces causes skin breakdown and abnormal bone deformation.

Compared to rigid braces, soft braces are compliant in nature. They prevent curve progression and, in some cases correct it, depending upon the severity of the Cobb angle. They can also be used to stabilize the spine after spine fusion surgery. Soft braces maximize exercise potential and improve the comfort and quality of life. SpineCor [34], a soft brace, was developed to overcome the drawbacks of rigid braces—specifically, problems of breathability, bulkiness, and physical constraints. SpineCor uses five elastic bands wrapped around the torso, which are attached to the contoured body west and pelvic waist. These multiple elastic bands apply three-dimensional (3D) corrective forces as the individual moves and generate more muscular balance. SpineCor is a full-time brace, and its recovery time depends on the severity of the spinal curve and the effect of the treatment itself [34]. Unlike its rigid counterparts, which result in spinal stiffness and muscle atrophy, SpineCor retains the overall posture by increasing muscle activity by strengthening the muscle around the spine. Despite its advantages, SpineCor is considered to be less effective in terms of curve correction compared to rigid braces [35].

Weiss developed a soft brace known as the SpinealiteTM brace, which differs from SpineCor in several aspects [36,37]. The corrective band used in SpinealiteTM is considerably stiffer than the SpineCor brace. Therefore, tensions of the band will remain uniform over time and corrective forces will remain steady, conversely to SpineCor’s unrestricted movements. A stiffer band makes SpinealiteTM comparatively less comfortable but brings more effective treatment results. This brace uses only one compression band to apply flexion corrective force on the sagittal plane and is suitable for treating lumbar lordosis.

A lateral force system TriaC brace was designed by Veldhuizen et al. [38]. It controls the rotation of the vertebral body by rotating the rear column to the concave side and the front column to the convex side. The effect of the correction in the frontal plane is similar to the rigid braces.

**Figure 2:**
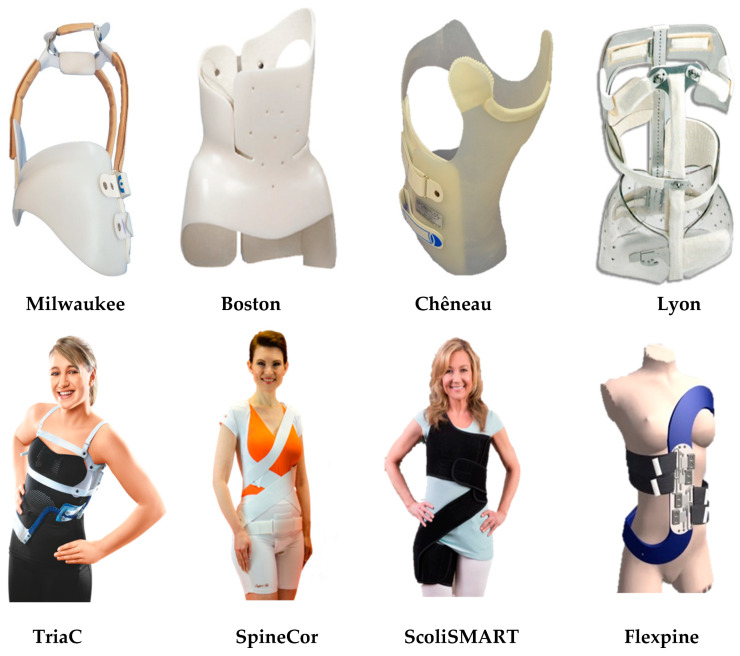
Rigid and soft corrective orthoses.

Several clinical results [39,40] have been described to assess the effect of a soft brace compared to rigid braces, but there is not enough evidence to deduce explicit conclusions regarding the effectiveness of the interventions [36]. Some of these braces can be seen in Figure 2. Table 1 describes the corrective orthoses and summarizes their key aspects, such as the brace name, developer country, year of development, rigidity, construction method and material, symmetry, the principle of correction, and targeted scoliosis curves. The objective is to describe the existing technologies in order to develop corrective orthoses and their applicability.

The current statistical studies [5,9,10] have determined that there is no adequate evidence to reach one concurrent decision about what type of brace is the best among all other types. The adequacy of the scoliosis treatment using braces remains controversial, due to inadequacy in the selection criteria of patients and the definition of brace efficacy. In order to compare the studies and validation of their reliability, the Scoliosis Research Society (SRS) has standardized criteria for the clinical trials of scoliosis patients with brace treatment. The SRS criteria include initial curve angles of 25–40°, age of 10 years or older at the time of brace prescription, no prior treatment, and being at Risser stages 0–2 [55]. The International Scientific Society on Scoliosis Orthopedic and Rehabilitation Treatment (SOSORT) produced its first guidelines in 2005, and renewed them in 2011 and 2016 to standardize them and align the use of braces and exercises into clinical practice of conservative treatment for idiopathic scoliosis (CTIS) [56].

## 4. Advancement in Spinal Rehabilitation Orthoses

A few researchers have tried to incorporate the technologies of the assistive orthoses into the corrective orthoses, to resolve several challenges faced by corrective orthoses, such as rigidity, lack of adjustability, higher construction time, sensorless design, and lack of force control over the specific vertebra.

### 4.1. Mobility and Actuation Technology

Mobility is key when it comes to treatment with braces. Conventional braces are rigid, passive, and do not allow mobility to the spine, which results in spine stiffness. Mobility can be achieved by either passive or active actuation. Actuation technologies in the area of assistive orthoses are quite matured with regard to achieving the goal of assisting the spine. Some of these actuation technologies have been introduced in corrective orthoses as well, such as the development of Spinecor and ScoliSMART, which are passive soft orthoses and use elastic material to apply corrective forces with the dynamic movement of the human body. Unfortunately, these passive braces do not provide force control over specific vertebra to apply the corrective forces.

**Figure 3:**
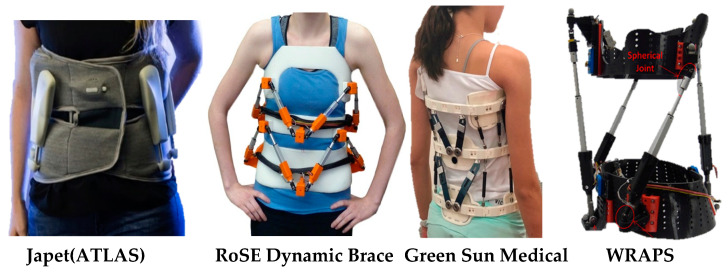
Smart rehabilitation orthoses for the spinal column.

To address the issues of mobility and apply controlled corrective forces over specific vertebra, the University of Colombia developed the RoSE dynamic brace. It uses two parallel Stewart platforms to apply forces in all directions, as shown in Figure 3. Forces and displacements applied by the RoSE can be measured by built-in pressure sensors and position sensors inside the actuator [17]. The RoSE exoskeleton is a big advancement in the area of active corrective orthoses, but has certain limitations. It uses eight series actuators that require a significant amount of power and also increase the device weight. This could be a limit in the use of RoSE, since these braces are supposed to be worn 18 h a day.

Spondylolisthesis is another type of spinal deformity, in which one of the vertebrae in the spine slips out of the proper position onto the vertebra below it, putting pressure on the nerve and a disc. Atlas Japet [13] developed an active disk decompression device that particularly helps to relieve people suffering from back pain caused by a herniated disk. Excessive vertebral compression of the intervertebral disks results in back pain. Atlas Japet alleviates the pain by applying distraction force on the vertebral column to increase the inter-joint space between vertebras. Exo-dynamics developed an active spinal brace called ExMS-1 [57], which offers automatic, customizable back support without restricting mobility. The goal of ExMS-1 is to prevent back pain from becoming serious spinal injury. 

Both Atlas Japet and ExMS-1 use series actuators that consume a lot of power. It is important to explore other actuation technologies that consume less power to reduce metabolic cost, as braces need to be worn for longer durations. One such mechanism is a twisted string actuation (TSA) [58]. TSA is translational transmission systems based on twisted strings coupled with electric motors, and results in lightweight, compact, and mechanically simple actuators [59]. TSAs are being used in a variety of wearable applications and have been used recently in spinal assistive devices [60]. Therefore, they have great potential to be used in the development of active dynamic braces.

### 4.2. Sensory Designs and Parameter Characterization

To treat a spinal deformity effectively, it is important to measure the physiological and mechanical parameters of the torso. Measuring the progression of the spine and adjusting the brace accordingly will expedite the process of recovery. Green Sun Medical developed a brace that provides physicians and patients with real-time performance metrics, utilizing a cloud-based health platform [61]. Measuring muscle activities would give feedback on muscle activation during the bracing time. This information is crucial from a physiotherapy point of view, in order to monitor the strengthening of the muscles. Myontec developed intelligent clothing technology to monitor the muscles’ activities, integrated with movement sensors for sports and rehabilitation purposes [62].

Outcomes of the brace treatment are associated with how effectively a brace is being worn. Force sensors and compliance monitors have been developed to monitor the quality of the brace usage. Thermobrace is a temperature sensor that is applied to the brace to monitor its actual wearing. It helps to optimize the therapies and helps doctors to understand the real effectiveness of the braces [11,63,64]. One of the key concerns in brace treatment is the lack of information on the forces that are being applied by the brace on the torso. Lou et al. [65] designed a wireless sensor network system to determine the biomechanics of spinal braces and continuously monitor the forces exerted by the brace on the spine. This system helps to examine the force distribution inside the brace during daily activities.

The effectiveness of the brace treatment depends on how the brace has been worn. It is important to wear the brace with the prescribed tightness to achieve a better outcome from the treatment. Lou et.al [66] developed an active intelligent brace system, which maintains the interface pressure in a prescribed range between the body and the brace. The brace uses an air bladder, which inflates to control the pressure between brace and body. An active intelligent brace increases the effective time of wearing the brace in prescribed tightness from 28% to 47% [66,67,68].

Braces correct the abnormal posture of the spine by applying several displacements at different levels of the torso. This is usually attained by adding soft pads or by adjusting the geometry of the brace design. These displacements result in corrective forces that are transmitted to the spine through soft tissues and the rib cage within the torso. Therefore, the amount of the curve correction depends on the stiffness of the torso, i.e., the stiffness of the soft tissues and stiffness of the spine. The stiffness characteristics of the torso may vary over time and during the course of the treatment, due to variation in the torso geometry, bone maturity spine curve, and fat distribution. Therefore, it is important to consider the torso stiffness characteristics along with the spine geometry to effectively design a brace. Park et al. [17] and Murray et al. [69] characterized torso stiffness in male and female patients using the RoSE dynamic brace.

Several other smart and active devices have been developed to characterize different physical parameters of the spine. These devices either help improve the scoliosis treatment or enhance the physiotherapy/training potential of the patients. Khan et al. [70,71] have developed the cable-driven trunk support trainer (TruST), which is helpful for the training of the seated posture for patients suffering from musculoskeletal and neurological disorders, where they have compromised postural stability. Based on a patient’s maximum trunk excursion, TruST control algorithms create a circular planar force tunnel around the trunk and provides as-needed assistance forces to the torso while performing the intended movements [72].

People with trunk impairment do not have enough strength in their trunk muscles to keep an upright posture or control the weight shifts to perform certain movements. Several passive orthoses are available to support the trunk by passively placing the torso and not allowing any degree of freedom to the trunk. Ophaswongse et al. [73] developed the wheelchair robot for active postural support (WRAPS). WRAPS supports the torso’s weight and is capable of replicating the patient’s trunk range of motion (tROM) without full activation of the trunk muscles. This is crucial for the people who do not have trunk control in antigravity postures, such as standing and sitting, when a reaching task is executed [74].

Table 2 describes the devices that are being used either to treat scoliosis effectively or for the training of the torso to enhance exercise potential. Several parameters, such as device name, actuation type, structure, application, and others, have been considered in the table.

### 4.3. CAD/CAM and Smart Materials

Three-dimensional printing revolutionized the conventional way of constructing braces. For decades, the conventional way to fabricate braces was by plaster cast, which involved manual measurements of the patient’s torso and designing a handmade brace. Technological revolution has enabled technicians to take body measurements using a photogrammetric scanning system, which is even faster than laser scanners [76]. Photogrammetric scanning systems have been proven to be effective for the fabrication of custom-made spinal orthoses, especially for patients with mobility impairments, as it allows them to capture instantaneously the torso shape with high accuracy (<1 mm) [77]. This allowed the fabrication of the CAD/CAM braces using 3D printing. These 3D-printed braces solve the socio-economic barrier of typical braces, and seem more appealing to the patients. Hence, the braces maximize patients’ willingness to wear a brace on a daily basis. It provides unparalleled personalization, incredible breathability, and reduces fabrication time. The Flexpine brace [52] is a semi-rigid brace used for conventionally treating scoliosis. It is 3D-printed brace, which uses 4 mm-thickn foldable plastic as its frame and elastic bands to apply corrective forces. It offers great mobility to the spine and overcomes the limitations of typical rigid braces. Various other braces, such as Boston, Chêneau, and Lyon, are also being 3D printed to enhance the breathability and reduce the fabrication time.

Several studies have demonstrated significant improvement in the results using CAD/CAM braces compared to the traditional approach, adding concrete scientific evidence (level of evidence II) [78,79,80]. However, these studies cannot offer a prior guarantee for better treatment results, as several other factors play a crucial role in brace treatment. Cobetto et al. [81,82], in two randomized controlled trials (RCTs), concluded that the combination of finite element modelling (FEM) and a CAD/CAM approach can further improve in-brace correction (IBC). The FEM braces exhibited 48% and 47% IBC for lumbar and thoracic curves, respectively, compared to 26% and 25% of CAD/CAM braces. Axial rotation correction of 46% compared to 30% by CAD/CAM braces. Moreover, the FEM braces were 50% thinner and had 20% less covering surface, making them more breathable for the wearer [81,82].

Various 3D printing techniques have been adapted in medical applications, such as stereolithography (SLA), Polyjet modeling (PJM), selective laser sintering (SLS), and fused deposition modeling (FDM). High equipment costs (especially for SLS and PJM) and processing times are the major limiting factors in the production of large orthotic devices using 3D printing. FDM is the most suitable and least expensive method to produce scoliosis braces among these 3D printing techniques, despite having a bit less dimensional accuracy. A few studies have recommended the use of 3D printing to construct orthotic devices [83,84,85]. The majority of the researchers have fixated on the feasibility of developing devices with accurate geometrical dimensions and desired shapes, but quite a few studies have reported the cost and mechanical performances of these devices. Furthermore, filament datasheets often refer to the mechanical properties of the bulk material before 3D printing, while the mechanical properties of the printed parts is inadequate and primarily focused on tensile properties [86]. Moreover, it is crucial to evaluate the toughness of the material through impact tests by measuring the energy absorbed for the duration of high strain rate conditions before failure. This behavior definitely varies with different production technologies, and the materials currently used for 3D printed orthotic devices do not provide the rigidity needed to correct the spine posture [85]. For these reasons, it is not yet possible to predict how the combination of virtual modeling and additive manufacturing processes affects the mechanical properties of a 3D-printed brace.

Since one of the major drawbacks of corrective orthoses is the uncomfortable rigidity that does not allow the necessary range of motion, the introduction of the principle of variable stiffness in design seems to be quite promising. This can be obtained either using new smart materials or developing specific design solutions. Smart materials have gained substantial consideration in medical applications. In particular, shape memory alloys (SMAs) are most generally employed for their super elasticity (SE) in orthopedic treatment. Chan et al. [87] developed a flexible scoliotic brace using SMAs. Among the suitable systems for variable stiffness, the jamming-based systems are emerging with a new set of possibilities [88,89]. Layer-jamming mechanisms have certain advantages, such as compactness, being lightweight, high resistance force, and fast reaction time. Jamming structures also possess the shape-locking capability, which can help to reduce the metabolic cost. They can be fabricated entirely using a 3D printing technique. Choi et al. [90] proposed that these structures can be used to develop the spinal assistance robot and in various other wearable applications.

## 5. Conclusions

The technologies presented in this article are a chunk of the bigger picture of improved spinal deformity treatment. Spinal deformity treatment remains an extremely qualitative process and relies heavily on the experience of the orthotist, physicians, physiotherapists, and patient compliance. The recommended developments will push spinal deformity treatment into more evidence-based practice. These technologies can be used to determine when bracing is most effective and how to improve the quality of life, as well as patient outcomes. This review article has focused on the following major areas:

1. Existing conventional braces and their key aspects, such as construction, materials, rigidity, and correction principle;
2. Advancement in brace spinal orthoses technologies in terms of mobility and actuation;
3. Use of sensors to track the brace compliance, interface pressure, force distribution, and torso parameter characterization;
4. Developments in brace construction technologies, such as CAD/CAM, 3D printing and smart materials.

Taking into consideration that these areas of research need to be streamlined and integrated, the direction of spinal deformity treatment needs to be not only the development of new kinds of braces and greater randomized control trials, but also the designing the technologies to supplement these braces. While technology in force and temperature sensors, as well as imaging and manufacturing methods, has progressed immensely, bracing has not taken advantage of this innovation. Prevalent implementation of these technologies will go a long way to successful patient outcomes from bracing for spinal deformities.

## Figures and Tables

**Table 1:** Corrective orthoses (braces and suits).

Device/Origin	Rigidity	Construction	Principle of Correction/Remarks
**Milwaukee brace, United States 1945** [14,18]	Rigid	Polyethylene, aluminum, and steel	Symmetrical design with a posterior opening. Previously used for post-operative immobilization of neuromuscular scoliosis. Not used anymore to treat scoliosis, but still used for Scheuermann’s kyphosis and high thoracic curves.
**Wilmington brace, United States 1969** [41]	Rigid	Polyethylene, custom-made/handmade	Thoracic-lumbar-sacral orthosis (TLSO) with underarm symmetrical design and anterior opening. Initially designed to treat curves between 25° and 39° with apices at or inferior to T7.
**Boston brace, United States 1972** [23,42]	Rigid	Polyethylene, prefabricated envelope/models	Symmetrical design with posterior opening. Developed for the lumbar curve, extended to treat thoracolumbar and thoracic curves. Reduced cost and fabrication time compared to Milwaukee. TLI (thoracolumbar lordotic intervention) by Loon et al. [37] to ensure forced lordosis at thoracolumbar spine. Applied when Cobb angle is over 25°
**Chêneau and derivatives, France/Germany1960** [31,43]	Rigid	Polyethylene, custom-made/CAD-CAM, handmade	The principle of correction of Chêneau brace is a combination of sagittal balance, regional de-rotation, physiological profile, and three-point pressure bending system. A three-dimensional (3D) Rigo System Chêneau brace (RSCB) and Chêneau light brace were developed as an extension of the J Chêneau brace in 1990 and 2005, respectively.
**Lyon brace,** **France 1947** [44,45]	Very rigid	Polymeta-crylate and radiolucent duralumin	The correction principle is the three-point pressure system with rotation angular breathing (RAB). Three regional, two-dimensional (2D) individual moldings. A 3D asymmetrical rigid torsion brace (ART), which is a Lyon brace derivative. Correction principle is global detorsion. Moldings: 3D helicoidal correction with coupled movements. Material: 4 m polycarbonate, rigid. The sagittal plane is fixed in a physiological posture to improve a flat back if necessary. In the middle, under the breast, the clamping of the two hemi-shells realizes the “tube mayonnaise” effect with passive axial lengthening and geometric detorsion. The polycarbonate–skin interface is a soft contact type with a mechanical detorsion of a cylinder.
**PASB, Italy 1976** [46]	Rigid	Polyethylene, custom/handmade	Progressive action short brace (PASB) is a TLSO for the correction of thoraco-lumbar, thoraco-lumbar-sacral, and idiopathic lumber curves.
**Charleston brace, United States 1979** [33,47]	Rigid	Polyethylene	Correction principle: Heuter–Volkmann principle TLSO, asymmetrical, anterior opening.Bending brace, side bending posture, single lumbar, thoracic, or thoracolumbar curves. Aggressive design for correction.
**Providence brace, United States 1992** [6]	Rigid	Polyethylene, custom-made/CAD-CAM, handmade	Surpasses the Charleston night brace due to less aggressive design. Asymmetric anterior opening. TLSO type, and curve correction by de-rotational and lateral forces as opposed to side bending posture, as seen in the Charleston brace. Very successful in treating flexible, single lumbar and thoracolumbar curves; however, it can be quite effective with thoracic and double curves.
**Dynamic Derotating Brace, Greece 1982** [48]	Rigid	Polypropylene and aluminum, custom made/CAD-CAM, handmade	Developed as a modification of the Boston brace in 1982, in Greece. It opens posteriorly, with a TLSO-type underarm brace with aluminum blades set to produce anti-rotating and de-rotating effects on the trunk and thorax of scoliosis patients.It is recommended for extremely high thoracic curves when the apex vertebra is T5 or above.
**Rosenberger brace, United States 1983** [49]	Rigid	Polyethylene	Correction principle: three-point pressure system.Asymmetrical, anterior opening, TLSO, reduces the curves with a translator and de-rotational loads. The limitation is its retrospective design.
**3D Sibilla brace** [50]	Low rigidity	−	Proposed for mild curve progression for a Cobb angle <30° that cannot be treated by SEAS * exercises. The brace is recommended to wear for 18 to 20 h daily, up to Risser stage 3.
**Sforzesco brace,** **Italy** [50]	Very rigid	Copolyester radiolucent duralumin, custom-made/CAD-CAM, handmade	3D active, symmetrical, incorporating the features of Milwaukee, Lyon, Sibilla, Risser cast, and Chêneau braces. Used for severe adolescent scoliosis (Cobb 45°–50°) when surgery is not a possible option or patients do not want it to be operated on. It is also a full-time brace and is recommended to be worn over 18 h a day.
**SpoRT Brace** [26,50]	Rigid	Polycarbonate, aluminum	The SPoRT bracing (three-dimensional elongation pushing in a down–up direction) is different from the other corrective systems: symmetric design, three-point, traction, and postural and movement-based.
**Jewett hyperextensionbrace,** [51]	Rigid	Metallic, prefabricated	Used to treat hyperkyphosis. It cannot handle rotational deformities of scoliosis. Stable framework construction restricts lateral flexion and hyperextension of the vertebral column, and provides stabilization in the sagittal plane.
**Flexpine brace, South Korea** [52]	Semi-rigid	3D-printed, elastic tissue, foldable plastic body	Lightweight, 4 mm-thick brace. 3D-printed brace made from foldable plastic. Allows mobility and enhances exercise’s potential to treat scoliosis.
**SpineCor dynamic brace, Canada 1993** [34,53]	Elastic	Elastic tissue, Prefabricated envelope/models	Dynamic bracing solution for idiopathic scoliosis and round back (hyperkyphosis) deformity. SpineCor treatment is suitable for children from the age of five with idiopathic scoliosis and certain syndrome-related scoliosis curves from 20°–50°. (Treatment is recommended for as low as 15° for children with a higher risk of progression.)
**SpinealiteTM brace** [36,37]	Elastic	Elastic tissue, prefabricated envelope/models	SpinealiteTM is used to treat lumbar lordosis. It uses a single band for the back compression force, which is quite helpful for the correction of flexion in the sagittal plane.
**Triac brace, Netherlands** [38,39]	Low rigidity	Soft plastic and metallic connections, prefabricated envelope/models	The flexible Triac brace was designed to improve cosmetic appearance and comfort. It was developed for primary curve correction in idiopathic scoliosis (IS). Planes of action are frontal and sagittal. Not recommended for the treatment of thoracic or double curves.
**ScoliSmart, USA** [54]	Soft suit	Prefabricated/fabric, elastic	ScoliSmart utilizes the energy of a human’s natural movement to generate new muscle memory. This new muscle memory decreases and stabilizes asymmetrical muscle firing, thus reducing the risk of curve progression and helping the spine unravel naturally, so it is never forced.

* SEAS: scientific exercises approach to scoliosis.

**Table 2:** Smart rehabilitation orthoses for the spinal column.

Device	Actuation	Structure	Application	Remarks
**Greensun medical brace, United States** [61]	Passive (elastic and metallic connections, prefabricated and adjusted for each patient)	Semi-rigid	Treat idiopathic scoliosis	It is a low-rigidity brace, consisting of semi-rigid segments encircling the torso, which are joined by the elastic elements. These elastic elements give required immobilization by engendering stabilizing forces while allowing the relative motion of semi-rigid segments. Real-time monitoring of the correction progress to adjust the brace.
**Inflatable intelligent active brace** [66]	Active (pneumatic bladder)	Rigid	Treat idiopathic Scoliosis	Use the air bladder to control interface pressure by inflating the bladder. The control system is comprised of a microcontroller, a force feedback component, and a force transducer.
**Japet** **(Atlas)** [75]	Active (four electric actuators)	Rigid	Pain relief, recover mobility	Extends the spine to release the pain. The adaptable system maintains complete freedom of movement without restricting muscular activity.
**ExMs-1 by Exo-dynamics** [57]	Active (four electric actuators)	Rigid	Pain relief, assistance while bending	Extends the spine and offers automatic, customizable back support without sacrificing mobility. This device is not intended to diagnose, treat, cure, or prevent any disease.
**RoSE dynamic brace** [17,69]	Active (electric, series elastic actuators)	Rigid (parallel Stewart platforms)	Treat idiopathic scoliosis, torso stiffness characterization	Three-point bending (push, movement, and elongation are other actuation mechanisms) and plane of action (3D, frontal, frontal horizontal, sagittal, and brace map classification).
**TruST** [70,71,72]	Active (electric, servo motors)	Rigid (pulley cable system)	Trunk support trainer	TruST is a cable pulley system; it uses four motors mounted on a stationary platform to apply forces through an adjustable but rigid belt on the trunk. TruST assists patients who have lost postural stability of the torso.
**WRAPS** [73,74]	Active series electric actuators	Rigid	Torso postural Support	WRAPS is a parallel robotic device consisting of two rings over the chest and hips connected by 2RPS-2UPS architecture. WRAPS can modulate forces/displacements applied to the torso in four degrees of freedom.

## Data Availability

Data available in a publicly accessible repository.
